# Community composition of zooplankton exported from a shallow polymictic reservoir linked to wind conditions

**DOI:** 10.7717/peerj.7611

**Published:** 2019-09-02

**Authors:** Nathan Ruhl, Desireé Haban, Caitlyn Czajkowski, Michael Grove, Courtney E. Richmond

**Affiliations:** Department of Biological Sciences, Rowan University, Glassboro, NJ, USA

**Keywords:** Zooplankton, Reservoir, Polymictic, Export, Wind

## Abstract

Zooplankton exported from lentic systems provision lotic systems with easily captured, consumed, and assimilated prey items. Previous studies have demonstrated that the community composition of zooplankton exports (CCZE) vary over time, which introduces temporal differences in lotic resource availability (zooplankton prey) in downstream habitats. In the study presented here, we monitored variation in CCZE from a polymictic reservoir outfall in response to physical–chemical and atmospheric conditions bi-hourly over three different 24-h periods. Community composition of zooplankton export varied over the course of the day, and exports were most closely associated with wind directionality. Future studies of temporal variation in CCZE should incorporate wind conditions, especially in shallow systems where holomixis occurs frequently. Polymictic reservoirs are becoming increasingly common as the global pace of small dam construction quickens, making both the identification of factors influencing CCZE and the impact of zooplankton exports on local biodiversity and ecosystem function increasingly important to understand.

## Introduction

Zooplankton are exported from lentic to lotic systems by water currents and the energy contained in their tissues can alter downstream resource availability (Akopian, Garnier & Pourriot, 1999), in turn altering ecosystem functions and the community structure of lotic consumers (Richardson, 1984; Richardson & Mackay, 1991; Brunke, 2004; Doi et al., 2008). Lotic filter-feeders can utilize a wide range of zooplankton types (Wotton, 1994), suggesting that the community composition of zooplankton exports (CCZE) is less important to lotic communities than abundance. This is a facile argument because zooplankton differ in the energy their bodies contain; larger zooplankters contain more energy (Walks & Cyr, 2004) and are more easily detected by visual predators (Brooks & Dodson, 1965). In fact, larger zooplankton are both preferentially (Chang et al., 2008; Czerniawski, Sługocki & Kowalska-Góralska, 2016) and rapidly (Czerniawski & Domagała, 2013) consumed downstream of lentic outlets, indicating that CCZE is important to the ecology of downstream consumers.

Zooplankton exports vary temporally at both seasonal (Portinho, Perbiche-Neves & Nogueira, 2016) and daily scales (Czerniawski, Sługocki & Kowalska-Góralska, 2016). Seasonal change in CCZE is likely to be the result of well-known patterns of seasonal succession in the plankton, but the mechanisms driving daily temporal variation in CCZE have not been fully explored. Zooplankton possess a number of behavioral adaptations including current avoidance (Fleminger & Clutter, 1965; Singarajah, 1969; Singarajah, 1975), shelter-seeking (horizontal migration; Burks et al., 2002; Walks & Cyr, 2004), and diel vertical migration (reviewed by Cohen & Forward (2009)) that could influence CCZE on a daily basis and all benefit zooplankters by increasing residence time in the lentic environment (Wicklum, 1999; Hülsmann & Wagner, 2007).

Humans are rapidly building new reservoirs in stream channels globally (Lehner et al., 2011), increasing the number of lotic/lentic transitions, which makes both the identification of factors influencing zooplankton exports and the impact of those exports on local biodiversity and ecosystem function increasingly important to understand. For zooplankton to be exported from lentic to lotic systems, adaptive behaviors need to be overcome, and an obvious mechanism by which that might occur is mixing. Mixing in lentic systems is usually associated with wind disturbance and is a function of the speed of the wind, strength/stability of vertical density gradients, depth, and fetch (Kalff, 2002). Mixing may also occur due to hydrological inputs that can vary from whole-system turnover (washout or “flushing”; Kalff, 2002) to isolated density interflows along the thalweg (Ford, 1990). Regardless of the mixing vector, mixing can cause zooplankton to be redistributed throughout the epilimnion (Hart, 1978), likely modifying zooplankton exports downstream.

In the study presented here, we monitored variation in CCZE bi-hourly over 24-h periods at a single location immediately below a reservoir outfall in response to atmospheric and physical–chemical conditions. We hypothesized that daily variation in CCZE would be structured by wind conditions.

## Methods

### Study site

**Figure 1:**
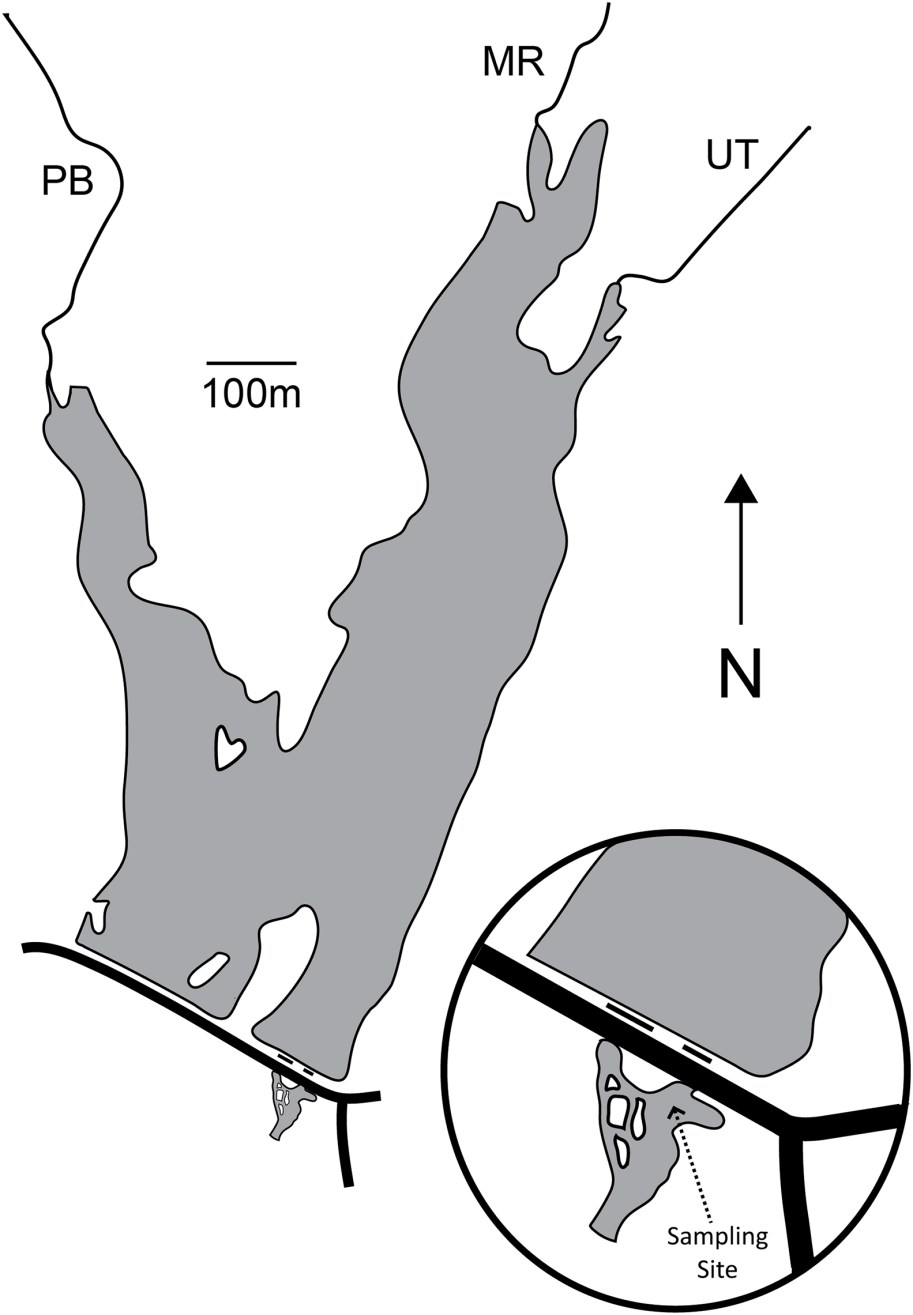
Map of Palatine Lake and the sampling location at the outfall confluence. There are two outfalls (short black lines) and a road (long black line) on the dam. Palatine Lake is fed primarily by Muddy Run (MR) and Palatine Branch (PB). An unnamed tributary (UT) drains a small watershed to the East of the lake.

Palatine Lake (39.541747°N, −75.169077°W; Fig. 1; Table 1) is a small polymictic reservoir located in Pittsgrove Township, Salem County, New Jersey (USA) that is owned and operated by the Palatine Lake Village Homeowners Association (PLVHA). The PLVHA gave us permission to use their facilities for this study and to sample the lake/outfall. Palatine Lake is fed primarily by Muddy Run and Palatine Branch, which together drain a watershed composed mostly of agricultural lands (53.0%) and wetlands (30.6%), but there are also relatively large areas of forest (7.2%) and urban (10.8%) habitat. Muddy Run continues downstream of Palatine Lake after passing through one of two outfalls on the dam, both of which are in continuous (concurrent) operation. All samples in the study presented here were taken from one site located in the confluence of the two outfalls (Fig. 1). The confluence pool has a natural bottom and edge that is pool-like, but the reservoir outfalls induce a strong mid-stream current that is run-like. Under base-flow conditions at the sampling location, the confluence has a width of 23.9 m, mean cross-sectional depth of 100.1 cm, and maximum cross-sectional depth of 158.5 cm. The maximum depth for the entire confluence is 214.0 cm.

**Table 1:** Palatine Lake characteristics.

Lake metric	Value
Watershed area (WA)	65.01 km^2^
Lake area (LA)	0.31 km^2^
WA:LA ratio	210:1
Volume	305,981 m^3^
Mean depth	1 m
Maximum depth	1.8 m
Length of shoreline	6.3 km
Maximum fetch	1,097 m
Operational fetch	305 m

### Field methods

Thirteen bi-hourly samples were collected during each of three 24-h periods: 12–13 June, 10–11 July, and 8–9 August 2017. Each bi-hourly sample is an integrated sample of five discrete casts of a bucket into the middle of the outfall stream (into the current) such that the benthos was not disturbed. The discrete casts were all collected within 5 min of each other and filtered through a 63-micron Nitex mesh. The total volume of water filtered for each bi-hourly sample was 38 liters. Samples were preserved with a stepped preparation of ~50% isopropyl alcohol in the field followed by further concentration and preservation in the lab to ~90%.

For each bi-hourly zooplankton sample, corresponding environmental data were collected either on-site or from a nearby environmental monitoring station (more details on specific sampling devices are given in Table 2). On-site atmospheric data were collected using a handheld anemometer measuring maximum wind speed (Variable: Maximum Gust) and air temperature. A variety of handheld meters were used to collect in-situ physical–chemical water quality data including dissolved oxygen (DO), water temperature, and pH. Conductivity, total dissolved solids, and turbidity were measured ex-situ within 20 min of sample collection.

**Table 2:** Environmental variables used to explain variation in CCZE.

Environmental variable	Data collection notes	Fitted units	Device/Sensor
Dissolved oxygen (DO)^a^	In-situ	mg/L	YSI 55
pH^a^	In-situ	log H+	YSI Ecosense pH100a
Conductivity^a^	Ex-situ (+20 min)	uS/cm	Eureka Manta 1
Total dissolved solids (TDS)^a^	Ex-situ (+20 min)	mg/L	Eureka Manta 1
Turbidity^a^	Ex-situ (+20 min)	NTU	Oakton T100
Water temperature^a^	In-situ	C	YSI Ecosense pH100a
Air temperature^a^	Unshaded temperature	C	Origlam Handheld Anemometer
Maximum gust^a^	3-min max	m/sec	Origlam Handheld Anemometer
Mean precipitation^b^	5-min mean	Mm	Texas Electronics TR-525I-HT
Mean solar radiation^b^	5-min mean	W/m^2^	Apogee SP110 Pyranometer
Total solar radiation^b^	5-min sum	W/m^2^	Apogee SP110 Pyranometer
Mean temperature^b^	5-min mean	C	Vaisala HMP45C
Count wind directions^b^	5-min max	# bearings	RM Young Company 05103
Mean wind directionality^b^	5-min mean	mean # bearings	RM Young Company 05103
Maximum wind speed^b^	5-min maximum	m/sec	RM Young Company 05103
Wind mean speed^b^	5-min mean	m/sec	RM Young Company 05103
Mean discharge^c^	Daily	ft^3^/sec	USGS Flood Gage
Maximum discharge^c^	Daily	ft^3^/sec	USGS Flood Gage

**Notes:**

a Measurements taken on-site at Palatine Lake concurrent with sampling.

b Measurements take off-site at weather station operated by Rutgers University (see text) 3.28 km SW of Palatine Lake. These variables were summarized at a variety of time lags before being fitted against the community ordinations. Time lags were 30 min concurrent with sampling and 30, 60, and 120 min prior to sampling.

c Measurements taken off-site at stream station operated by USGS 9.4 km SE of Palatine Lake. These variables were summarized for the week leading up to sampling.

Off-site weather data were obtained from a weather station located 3.28 km to the southwest (39.524351°N, −75.200927°W) of our sampling location; the weather station is operated by the Rutgers University Agricultural Extension (Upper Deerfield, NJ Mesonet Station; https://www.njweather.org/station/284). The weather station variables we used were precipitation, total solar radiation, mean solar radiation, wind speed, and wind direction (Table 2). Numerical weather station data were collected at 5-min intervals, binned at a variety of time-periods relative to when on-site zooplankton sampling occurred (30 min concurrent with sampling and at 30, 60, and 120 min leading up to zooplankton sampling), and summarized (maximum, mean, or sum). Binning of data at a variety of time lags was done to account for possible discontinuity in timing between environmental conditions at the weather station and the response of zooplankton exports to changing conditions.

Wind direction data (categorical headings) obtained from the weather station were reported as a single direction (1 of 16 possible directions) for a 5-min period (a 5-min mean). These categorical wind directions were transformed to measures of variability in the direction of the wind (directionality) by expressing the data as (1) a count of how many different wind directions were observed prior to sampling (Maximum = 16; Variable: Count Wind Directions, Table 2) and (2) as a count of how many different wind directions were observed prior to sampling divided by the number of observations (Variable: Mean Wind Directionality, Table 2). The number of observations (5-min means) varied by the time period being considered (binning by 30, 60, or 120 min). For both wind directionality variables, higher values indicate winds blowing from an inconsistent direction whereas lower values indicate the wind is blowing from a consistent direction.

Hydrological data were collected from a USGS stream gauge in an adjacent watershed 9.4 km to the southeast (39.495750°N, −75.076496°W) of our sampling location; these data include mean and maximum daily discharge (given as ft^3^/second and fitted as m^3^/second) for the week (7 days) leading up to sampling.

### Lab methods

Field-preserved samples were concentrated using a 63-micron Nitex mesh and preserved at a final concentration of ~90% isopropyl alcohol. The water volume filtered (38 L) and the final concentrated sample were standardized across all samples (*n* = 39; 13 bi-hourly samples per 24-h period) to allow for comparisons of abundance between samples. Zooplankton were identified according to Haney (2019) under 40× magnification using a one mL Sedgwick-Rafter counting chamber that was loaded from a well-mixed sample (McCauley, 1984). For samples with low abundance, the sub-sample was drawn entirely from the sample (one mL of mixed sample directly into the counting chamber). For samples with high abundance, the sub-sample was diluted in the counting chamber (e.g. 1:1 = 0.5 mL sub-sample + 0.5 mL isopropyl alcohol). For each sample, at least six sub-samples were counted. Dilutions of 1:1 and 1:3 were typical dilutions (maximum dilution 1:20 for one sample). In the final analysis density was standardized to individuals/liter.

Zooplankton were identified to the following taxonomic groups: copepod nauplii (Naup), adult copepods and copepodites (Cop), *Bosmina* (Bos; a cladoceran genus), Chydorids (Chy; a cladoceran family), other cladocerans (Clad), rotifers (Rot), midges (Midge; *Chaoborus*, a genus of planktivorous midge larvae that are members of the zooplankton), and ostracod (Ostra). These taxonomic groups were chosen a priori based on a combination of factors including confidence of taxonomic identification at 40× magnification, minimizing the undue influence of rare species on ordinations, and on known behavioral/ecological responses to environmental conditions.

### Statistical methods

The role of environmental variables in structuring CCZE was explored via ordination of the export community (indirect gradient analysis approach; non-metric multi-dimensional scaling (NMDS); metaMDS function in the Vegan package of R; Bray–Curtis dissimilarity). For each of the three 24-h periods, temporal shifts in CCZE were visualized by plotting bi-hourly samples with an NMDS ordination and then vector-fitting environmental variables to the underlying ordination to independently assess their predictive power for CCZE. In these analyses (for each 24-h period), space is held as constant (the same location is repeatedly sampled) and time is assumed to vary (points in the ordination diverge because of differences in time). Environmental variables (Table 2) were fit to the NMDS ordination using the envfit function in the Vegan package of R. Some turbidity values during 10–11 July as well as conductivity and TDS values during 8–9 August were not useable, so these variables were not fitted to the corresponding 24-h ordinations or to the combined seasonal (comparing 24-h periods) ordination detailed below. The hydrological data were not fit to NMDS ordinations because of the potential for discontinuity between measurements from a different watershed at nearly 10 km distance from the location where CCZE was being assessed. Likewise, species vectors are not displayed in the NMDS ordinations because it is difficult to interpret species vectors relative to environmental vectors in an unconstrained ordination.

Seasonal (between sampling period) differences in the CCZE were explored for each taxonomic group via Kruskal–Wallis test by ranks with Steel-Dwass pairwise tests (JMP 13, SAS Institute) and a NMDS ordination using all bi-hourly samples (all three 24-h sampling periods combined; R). This ordination contrasts samples taken at two different time scales (bi-hourly and monthly across 24-h sampling periods) and has utility for assessing differences in CCZE between months (factor-fit). The fit of environmental variables to this ordination was assessed conservatively using α = 0.001 instead of 0.05 to avoid a type 1 error associated with using data collected at multiple scales in the same ordination. Comparison of hydrological data between 24-h sampling events (months) was conducted using Kruskal–Wallis test by ranks and Steel-Dwass pairwise tests, α = 0.05.

## Results

### Fit of environmental variables to CCZE

**Figure 2:**
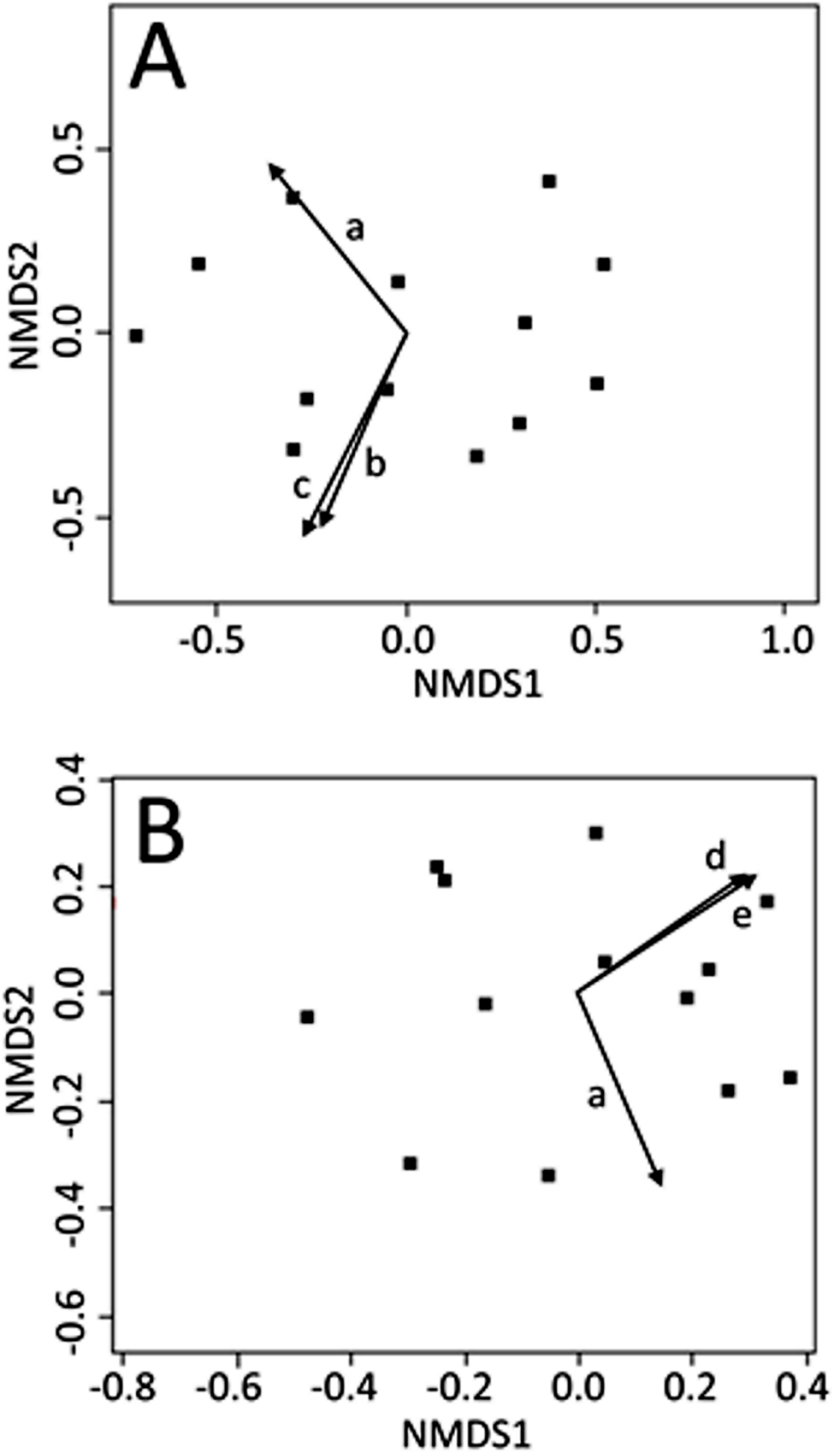
NMDS bi-plots of zooplankton export. NMDS bi-plots of zooplankton exports during 12–13 June (A) and 10–11 July (B) with significant environmental variables fitted to the ordination as vectors. Significant environmental vectors were mean wind directionality measured concurrently with zooplankton sampling (a) and during the 60 (b) and 120 (c) min leading up to zooplankton sampling as well as count wind directions in the 30 (d) and 120 (e) min leading up to zooplankton sampling. Fitting statistics are given in Appendix 1 and the text.

Non-metric multi-dimensional scaling ordinations were plotted in two dimensions (stress: June = 0.112; July = 0.106; August = 0.119) and environmental explanatory variables were fit to the underlying ordination (see Appendix Table A1, for fitting statistics and *p*-values for all environmental variables). No light variables were significant predictors of CCZE during any 24-h period (June, July, or August). Mean wind directionality measured concurrently with on-site zooplankton sampling was correlated with the zooplankton community ordinations in both June (Fig. 2A, *r*^2^ = 0.535, *p* = 0.023) and July (Fig. 2B, *r*^2^ = 0.510, *p* = 0.031). Additional wind directionality variables were significantly correlated with the June ordination (Fig. 2A; 60-min mean wind directionality, *r*^2^ = 0.496, *p* = 0.031; 120-min mean wind directionality, *r*^2^ = 0.574, *p* = 0.012) and July ordination (Fig. 2B; 30-min count wind directions, *r*^2^ = 0.476, *p* = 0.039; 120-min count wind directions, *r*^2^ = 0.515, *p* = 0.029). No measured environmental variables were significant predictors of CCZE in August.

### Differences in zooplankton exports between 24-h periods (months)

Zooplankton density (all taxa of zooplankton combined) was highest in July (significantly higher in July than in June, *p* = 0.008), but was highly variable between samples and between 24-h sampling periods (months; Tables 3 and 4). Naupliar density increased significantly across months while midge density decreased. Peak density for most groups occurred in July; some groups (*Bosmina* and cladoceran) were significantly more common in July samples than in either June or August samples (*p* < 0.001). Copepods significantly increased in density from June to July but, while density was much lower in August than in July, this decrease was only marginally significant (*p* = 0.079). Midge and ostracod density decreased significantly between the July and August sampling periods, while rotifer and chydorid groups did not differ between months.

**Table 3:** Mean bi-hourly abundance (density; ind./l lake water) and SD of zooplankton during each 24-h sampling period.

Group	June		July		August	
	Mean	SD	Mean	SD	Mean	SD
Nauplii	4.751	2.465	36.984	11.242	49.644	20.107
Copepod	1.899	2.757	15.505	18.229	2.560	2.829
*Bosmina*	0.526	0.820	89.082	46.809	0.213	0.554
Chydorid	0.434	0.667	13.513	2.346	0.142	0.347
Cladoceran	0.341	1.018	6.935	7.315	0	0
Rotifer	175.895	133.257	365.005	547.424	307.110	361.392
Midge	0.605	0.605	0.356	0.601	0	0
Ostracod	4.296	4.794	6.259	5.354	1.849	1.645
All groups	188.747	134.075	521.476	583.128	361.520	372.404

**Note:**

“All groups” give the mean abundance (density) and SD of zooplankters in bi-hourly samples from all taxonomic groups combined.

**Table 4:** Results of a non-parametric multiple comparisons analysis (Steel-Dwass) of exported zooplankton density (bi-hourly abundance) for each taxonomic group between 24-h periods (months).

Group	June–July		June–August		July–August	
	*Z*	*p*	*Z*	*p*	*Z*	*p*
Nauplii	4.311	**<0.001**	4.312	**<0.001**	1.898	**0.139**
Copepod	2.532	0.031	1.178	0.466	2.158	0.079
*Bosmina*	4.371	**<0.001**	1.248	0.425	4.479	**<0.001**
Chydorid	0.088	0.996	1.146	0.486	1.476	0.303
Cladoceran	3.871	**<0.001**	1.757	0.184	4.329	**<0.001**
Rotifer	1.692	0.208	1.487	0.297	0.667	0.783
Midge	1.153	0.482	2.086	0.093	3.234	**0.004**
Ostracod	1.361	0.361	1.116	0.504	2.589	**0.026**
All groups	2.974	**0.008**	2.051	0.100	2.154	0.079

**Note:**

*Z* is reported as the absolute value. Significant differences are indicated in bold. **“**All groups” give the mean abundance (density) and SD of zooplankters in bi-hourly samples from all taxonomic groups combined.

**Figure 3:**
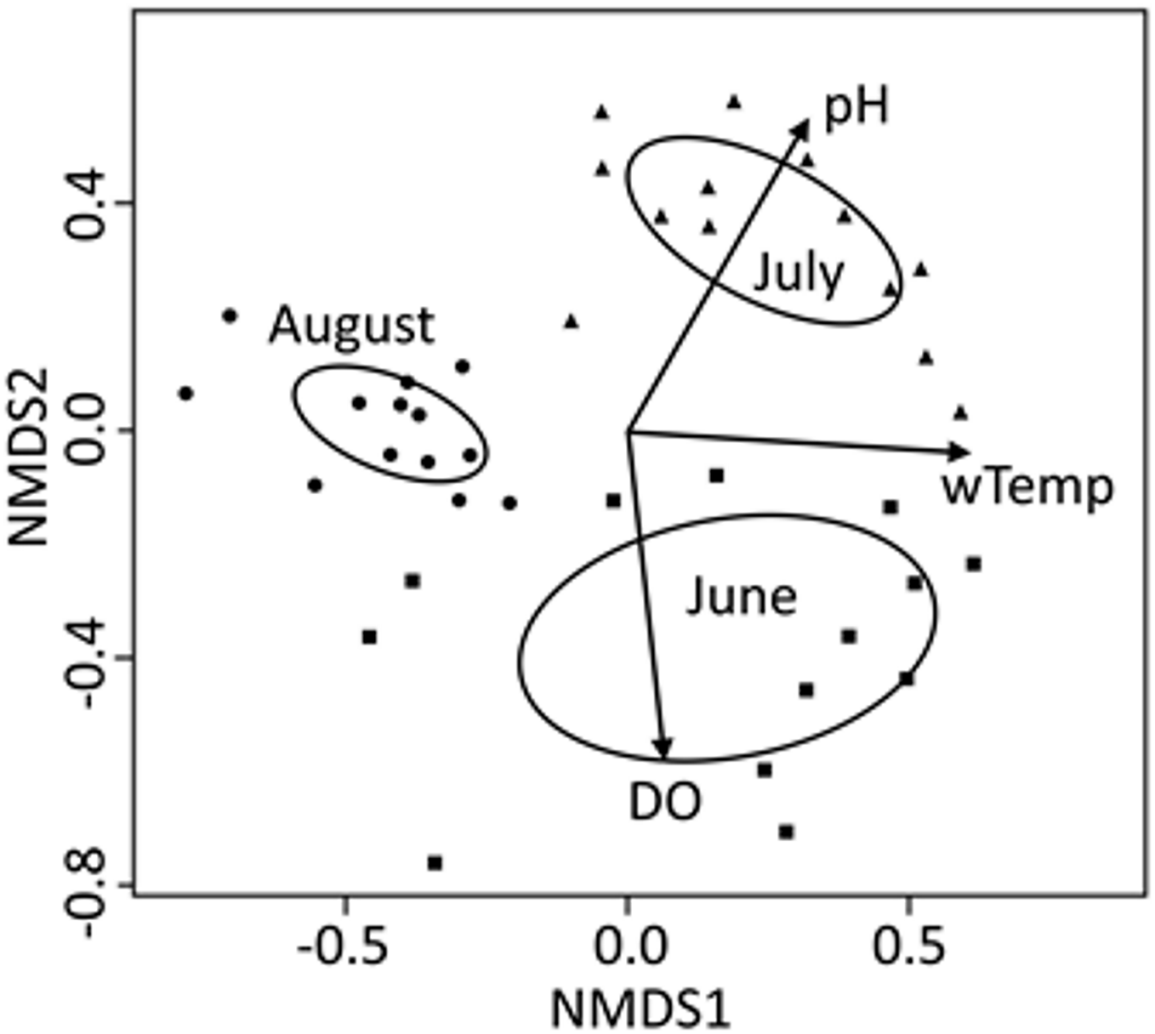
NMDS bi-plot for all three 24-h sampling periods (months) combined. Variables from Table 2 with a significant fit (*p* < 0.001; Appendix 1) are shown. Ellipses represent 95% confidence of factor (month) centroids. Samples from June are indicated with squares, July with triangles, and August with circles.

**Figure 4:**
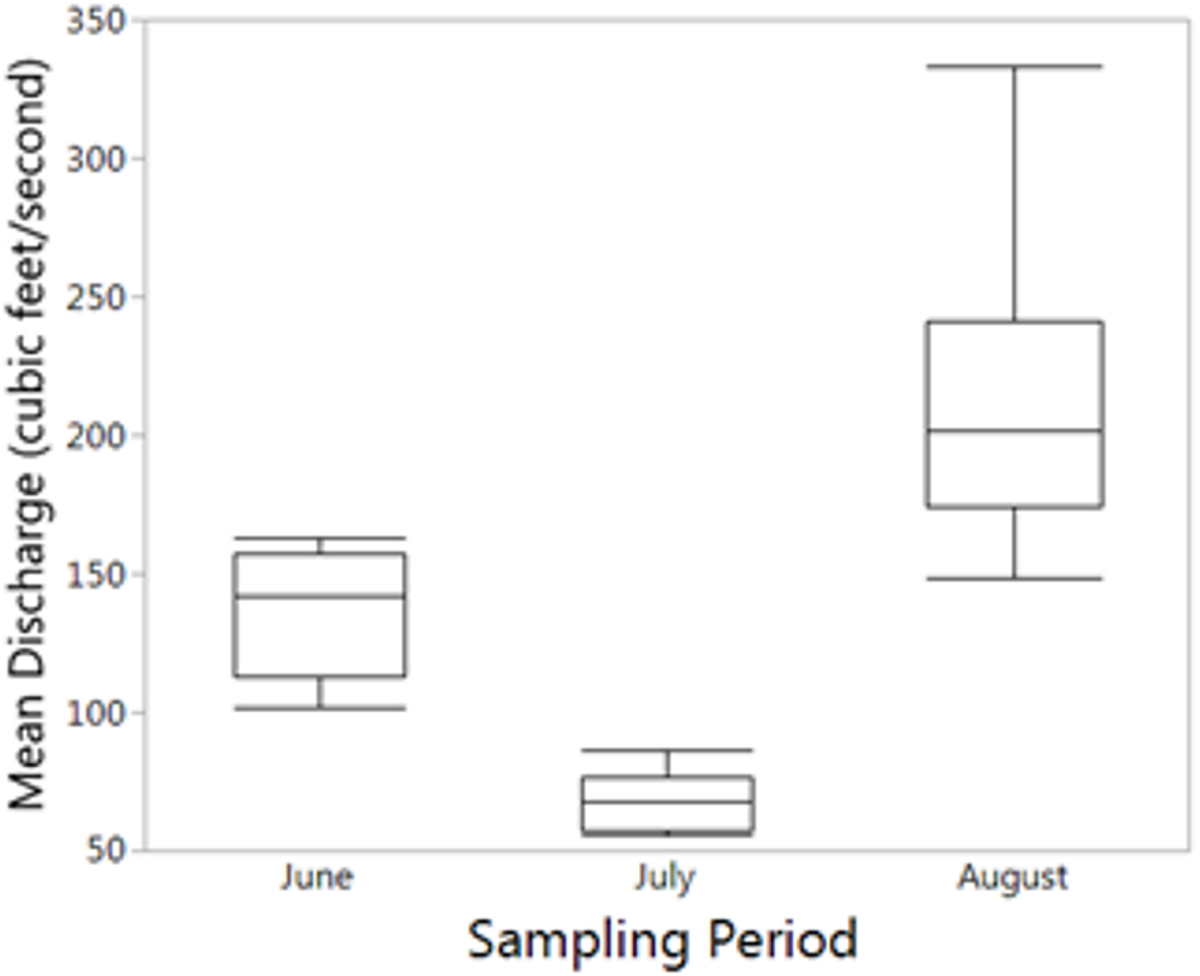
Mean reservoir discharge. Mean reservoir discharge leading up to sampling was significantly different between all months (Kruskal–Wallis: *p* < 0.001).

For the seasonal (all months combined) NMDS ordination (stress = 0.165), CCZE varied significantly between months (Appendix 1 and Fig. 3; months, *r*^2^ = 0.652, *p* = 0.001). A few environmental variables were significant predictors of the NMDS ordination at the alpha = 0.05 level, but only physical–chemical parameters were significant at an adjusted alpha of 0.001 (DO, *r*^2^ = 0.320, *p* < 0.001; pH, *r*^2^ = 0.397, *p* < 0.001; water temperature, *r*^2^ = 0.356, *p* < 0.001). Hydrological conditions were significantly different between monthly 24-h sampling events (Fig. 4; Kruskal–Wallis comparing 24-h sampling period between months, *p* < 0.001; absolute value of *Z* in Steel-Dwass test; June–July: *Z* = 3.066, *p* = 0.006; June–August: *Z* = 2.814, *p* = 0.014; July–August: *Z* = 3.06998, *p* = 0.006).

## Discussion

Our results indicate that wind variables were significantly correlated with the underlying community structure in the NMDS ordinations (Fig. 2), suggesting CCZE may be structured by wind-generated mixing in a shallow polymictic reservoir. To the best of our knowledge, this is the first time that CCZE has been linked to wind conditions. If wind disturbance modulates CCZE, then the susceptibility of a given lentic system to wind mixing likely influences lotic resource availability and introduces variation in lotic ecosystem function downstream of lentic outlets/outfalls.

Wind speed may be less important to governing CCZE than variation in the direction of the wind. Mean wind directionality measured concurrently with zooplankton sampling was significantly correlated to the underlying NMDS ordinations in both June and July, suggesting that zooplankton exports are structured along a gradient from straight-line to swirling winds. When wind directionality is low, the wind is blowing consistently in the same direction, which causes mixing forces to be reinforced to depth (Kalff, 2002). When wind directionality is high, the wind is swirling and blowing from an inconsistent direction, which may cause mixing forces to be canceled out, and mixing may not occur to depth.

Determining which wind variable(s) was most responsible for influencing community composition is not straightforward and requires additional study. While the distance between our sampling site and the weather station is not excessive and there are no major topographic features (e.g. hills) to distort wind patterns in our study area, wind conditions can vary greatly between nearby locations (e.g. Mahrt, 2010), so future studies would benefit from an even closer pairing of zooplankton export data with on-site weather data and instantaneous compass bearings.

Community composition of zooplankton export was significantly different between the three different time periods of this study and physical–chemical variables (pH, water temperature, and DO) were very strong explainers of those monthly differences as evidenced by the consistency between ellipses and vectors in the combined NMDS analysis (Fig. 3). Environmental variables cannot be used as response variables in this ordination due to a disjunct in temporal distance between samples (bi-hourly or monthly), but they can be used to differentiate between monthly classes of samples. August stands out as different because some taxa that were present in June and July were no longer present in exports (Table 3; other cladocerans and midges). Seasonal changes in the zooplankton community are likely a natural feature of this reservoir (e.g. Sommer et al., 1986), but the lake-wide application of herbicide to control macrophytes and limited littoral macrophyte raking, which occurred between our July and August sampling, may help explain why cladoceran and planktonic midge abundance was different in August. Negligible numbers of planktonic midges during our August sampling could also have been caused by these larvae emerging from the reservoir before our sampling took place.

Hydrological disturbance (Dickerson, Medley & Havel, 2010) and water residence time (Jann & Bürgi, 1988; Burdis & Hirsch, 2017) have been linked to CCZE and may cause a phenomenon known as “washout” whereby community composition is shifted toward quickly reproducing taxa (rotifers) that can complete their life cycle before being exported (Lair, 2006; Perbiche-Neves & Nogueira, 2013). In our study, hydrological monitoring was at a comparatively coarse scale (daily mean discharge), so we could not determine how flow rate influenced our bi-hourly zooplankton samples. We chose to study a polymictic reservoir because we felt that this type of lentic habitat was likely to exhibit a wind-induced effect on community composition; natural lakes and lentic systems that mix less frequently (e.g. dimictic lakes) might not exhibit wind-induced shifts in CCZE. Future studies of zooplankton exports from deeper (more stably stratified) lentic systems should directly monitor the thermal stratification regime in the pool.

In this study we did not detect a significant effect of light on CCZE, indicating that diel migration was less important to structuring zooplankton exports than other forces (i.e. wind). Diel migrations may be important factors in temporally structuring CCZE in shallow systems that are not readily mixed by wind (e.g. canals), but we have little evidence to suggest that diel migrations contributed to structuring exports in this study. We used a coarse taxonomy to define CCZE in this study, so further study of the importance of diel and wind factors on zooplankton export should explore whether the same patterns occur at finer taxonomic scales and how different taxonomic groups are independently influenced by wind mixing (i.e. constrained ordination).

Our results suggest that wind conditions could introduce temporal heterogeneity in food resources to downstream communities which, in turn, should promote biodiversity among consumers (Tylianakis et al., 2008; but see Cardinale et al., 2006). Conversely, homogenization of food resources should promote competitive exclusion and a decrease in biodiversity, which is the effect that reservoirs are generally thought to have on downstream communities (Kalff, 2002). Small polymictic reservoirs may therefore not have the same negative impacts that less-frequently mixed (deeper) or washed-out (higher flow) reservoirs have on downstream communities. Identification of mechanisms impacting zooplankton export from lentic to lotic systems is increasingly important as the number of reservoirs continues to grow globally (Lehner et al., 2011).

## Supplemental Information

10.7717/peerj.7611/supp-1 Supplemental Information 1 Fitting statistics (*r*^2^ and *p*-value) to NMDS ordinations for each monthly 24-h sampling period and for all three sampling periods combined (all months). **Notes:** Significant *p*-values and accompanying *r*^2^ values are indicated in bold. Alpha = 0.05 for single month ordinations. Alpha = 0.001 for all months combined. ^a^: Incomplete data due to equipment failures ^b^: Sampling did not coincide with appreciable precipitation.

10.7717/peerj.7611/supp-2 Supplemental Information 2 Zooplankton, physical–chemical, and atmospheric data for this study. Data are arranged by data and sample order. The first sample was at noon and samples are bi-hourly after that. Zooplankton abundances are scaled to individuals per liter. Units for other variables can be found in the text.

## Additional Information and Declarations

### Field Study Permissions

The following information was supplied relating to field study approvals (i.e., approving body and any reference numbers): The state of NJ does not required a scientific sampling permit for zooplankton or water quality monitoring. Palatine Lake is privately owned and we received permission to sample the lake from the homeowner’s association.

### Data Availability

The following information was supplied regarding data availability: The raw data are available as a Supplemental File. The data used in this study include zooplankton and physicochemical data obtained at the Outfall site (O) draining Palatine Lake and weather station data from the Upper Deerfield NJ station.
