# Supplementary material for: Community composition of zooplankton exported from a shallow polymictic reservoir linked to wind conditions
Source: PeerJ. 2019 Sep 2;7:e7611. doi: 10.7717/peerj.7611 (PMC6727831; doi:10.7717/peerj.7611)
Supplement: Supplemental Information 1 — Notes: Significant p-values and accompanying r2 values are indicated in bold. Alpha = 0.05 for single month ordinations. Alpha = 0.001 for all months combined. a: Incomplete data due to equipment failures b: Sampling did not coincide with appreciable precipitation. [file peerj-07-7611-s001.docx]

**Appendix**

**Table A1:** Table of fitting statistics (r^2^ and p-value) to NMDS ordinations for each monthly 24-hour sampling period and for all three sampling periods combined (all months).

|  |  | **June** | | **July** | | **August** | | **All Months** | |
| --- | --- | --- | --- | --- | --- | --- | --- | --- | --- |
| **Time Lag** | **Variable** | **r^2^** | **p** | **r^2^** | **p** | **r^2^** | **p** | **r^2^** | **p** |
| Concurrent | Dissolved Oxygen | 0.047 | 0.832 | 0.060 | 0.714 | 0.346 | 0.120 | **0.320** | **<0.001** |
|  | pH | 0.093 | 0.628 | 0.072 | 0.676 | 0.156 | 0.428 | **0.397** | **<0.001** |
|  | Conductivity | 0.193 | 0.377 | 0.170 | 0.400 | ^a^N/A | ^a^N/A | ^a^N/A | ^a^N/A |
|  | Total Dissolved Solids | 0.197 | 0.362 | 0.126 | 0.498 | ^a^N/A | ^a^N/A | ^a^N/A | ^a^N/A |
|  | Turbidity | 0.183 | 0.374 | ^a^N/A | ^a^N/A | 0.198 | 0.339 | ^a^N/A | ^a^N/A |
|  | Water Temperature | 0.189 | 0.355 | 0.070 | 0.686 | 0.083 | 0.651 | **0.356** | **<0.001** |
|  | Air Temperature | 0.024 | 0.886 | 0.233 | 0.274 | 0.111 | 0.556 | 0.065 | 0.301 |
|  | Maximum Gust | 0.110 | 0.570 | 0.082 | 0.651 | 0.118 | 0.532 | 0.038 | 0.507 |
| Concurrent 30 minutes | Mean Precipitation | ^b^N/A | ^b^N/A | ^b^N/A | ^b^N/A | ^b^N/A | ^b^N/A | ^b^N/A | ^b^N/A |
|  | Mean Solar Radiation | 0.351 | 0.114 | 0.181 | 0.367 | 0.198 | 0.335 | 0.062 | 0.308 |
|  | Total Solar Radiation | 0.351 | 0.115 | 0.170 | 0.396 | 0.191 | 0.349 | 0.062 | 0.312 |
|  | Mean Temperature | 0.235 | 0.256 | 0.177 | 0.369 | 0.135 | 0.488 | 0.119 | 0.102 |
|  | Count Wind Directions | 0.285 | 0.190 | 0.318 | 0.147 | 0.165 | 0.407 | 0.276 | 0.002 |
|  | Mean Wind Directionality | **0.535** | **0.023** | **0.510** | **0.031** | 0.192 | 0.346 | 0.155 | 0.046 |
|  | Maximum Wind Speed | 0.088 | 0.627 | 0.171 | 0.395 | 0.210 | 0.306 | 0.173 | 0.033 |
|  | Mean Wind Speed | 0.020 | 0.895 | 0.267 | 0.213 | 0.229 | 0.270 | 0.246 | 0.008 |
| Prior 30 minutes | Mean Precipitation | ^b^N/A | ^b^N/A | ^b^N/A | ^b^N/A | ^b^N/A | ^b^N/A | ^b^N/A | ^b^N/A |
|  | Mean Solar Radiation | 0.284 | 0.181 | 0.204 | 0.313 | 0.233 | 0.269 | 0.064 | 0.300 |
|  | Total Solar Radiation | 0.281 | 0.185 | 0.221 | 0.281 | 0.243 | 0.252 | 0.063 | 0.304 |
|  | Mean Temperature | 0.188 | 0.347 | 0.107 | 0.557 | 0.141 | 0.472 | 0.129 | 0.083 |
|  | Count Wind Directions | 0.039 | 0.825 | **0.476** | **0.039** | 0.033 | 0.849 | 0.053 | 0.377 |
|  | Mean Wind Directionality | 0.355 | 0.119 | 0.249 | 0.237 | 0.076 | 0.676 | 0.162 | 0.037 |
|  | Maximum Wind Speed | 0.102 | 0.566 | 0.281 | 0.198 | 0.136 | 0.475 | 0.098 | 0.155 |
|  | Mean Wind Speed | 0.037 | 0.820 | 0.351 | 0.119 | 0.157 | 0.421 | 0.128 | 0.086 |
| Prior 60 minutes | Mean Precipitation | ^b^N/A | ^b^N/A | ^b^N/A | ^b^N/A | ^b^N/A | ^b^N/A | ^b^N/A | ^b^N/A |
|  | Mean Solar Radiation | 0.274 | 0.195 | 0.190 | 0.336 | 0.213 | 0.305 | 0.073 | 0.249 |
|  | Total Solar Radiation | 0.272 | 0.198 | 0.198 | 0.320 | 0.217 | 0.295 | 0.072 | 0.252 |
|  | Mean Temperature | 0.171 | 0.385 | 0.084 | 0.634 | 0.143 | 0.462 | 0.132 | 0.077 |
|  | Count Wind Directions | 0.241 | 0.246 | 0.370 | 0.093 | 0.042 | 0.811 | 0.087 | 0.191 |
|  | Mean Wind Directionality | **0.496** | **0.031** | 0.113 | 0.548 | 0.017 | 0.915 | 0.217 | 0.010 |
|  | Maximum Wind Speed | 0.051 | 0.764 | 0.133 | 0.493 | 0.100 | 0.584 | 0.102 | 0.145 |
|  | Mean Wind Speed | 0.030 | 0.850 | 0.215 | 0.293 | 0.138 | 0.468 | 0.119 | 0.105 |
| Prior 120  Minutes | Mean Precipitation | ^b^N/A | ^b^N/A | ^b^N/A | ^b^N/A | ^b^N/A | ^b^N/A | ^b^N/A | ^b^N/A |
|  | Mean Solar Radiation | 0.286 | 0.182 | 0.157 | 0.418 | 0.191 | 0.347 | 0.072 | 0.253 |
|  | Total Solar Radiation | 0.283 | 0.187 | 0.160 | 0.411 | 0.195 | 0.338 | 0.071 | 0.258 |
|  | Mean Temperature | 0.142 | 0.459 | 0.080 | 0.650 | 0.148 | 0.452 | 0.149 | 0.054 |
|  | Count Wind Directions | 0.146 | 0.460 | **0.515** | **0.029** | 0.090 | 0.627 | 0.131 | 0.081 |
|  | Mean Wind Directionality | **0.574** | **0.012** | 0.087 | 0.628 | 0.033 | 0.840 | 0.234 | 0.007 |
|  | Maximum Wind Speed | 0.057 | 0.741 | 0.098 | 0.603 | 0.050 | 0.766 | 0.086 | 0.194 |
|  | Mean Wind Speed | 0.029 | 0.863 | 0.059 | 0.740 | 0.077 | 0.662 | 0.129 | 0.084 |

**Notes:** Significant p-values and accompanying r^2^ values are indicated in bold. Alpha=0.05 for single month ordinations. Alpha=0.001 for all months combined.

^a^: Incomplete data due to equipment failures

^b^: Sampling did not coincide with appreciable precipitation
